# Human Influenza A(H7N9) Virus Infection Associated with Poultry Farm, Northeastern China

**DOI:** 10.3201/eid2011.140608

**Published:** 2014-11

**Authors:** Ming Fan, Biao Huang, Ao Wang, Liquan Deng, Donglin Wu, Xinrong Lu, Qinglong Zhao, Shuang Xu, Fiona Havers, Yanhui Wang, Jing Wu, Yuan Yin, Bingxin Sun, Jianyi Yao, Nijuan Xiang

**Affiliations:** Jilin Provincial Center for Disease Control and Prevention, Jilin, China (M. Fan, B. Huang, A. Wang, L. Deng, D. Wu, X. Lu, Q. Zhao, S. Xu);; Changchun Prefectural Center for Disease Control and Prevention, Jilin (Y. Wang, J. Wu, Y. Yin, B. Sun);; Chinese Center for Disease Control and Prevention, Beijing, China (J. Yao, N. Xiang);; Centers for Disease Control and Prevention, Atlanta, Georgia, USA (F. Havers)

**Keywords:** H7N9, avian influenza, China, poultry farm, influenza, flu, avian flu, human, *Suggested citation for this article*: Fan M, Huang B, Wang A, Deng L, Wu D, Lu X, et al. Human influenza A(H7N9) virus infection associated with poultry farm, northeastern China. Emerg Infect Dis [Internet]. 2014 Nov [*date cited*]. http://dx.doi.org/10.3201/eid2011.140608

## Abstract

We report on a case of human infection with influenza A(H7N9) virus in Jilin Province in northeastern China. This case was associated with a poultry farm rather than a live bird market, which may point to a new focus for public health surveillance and interventions in this evolving outbreak.

Since it was first reported in spring 2013 (*1*), influenza A(H7N9) virus has caused 436 confirmed human infections, resulting in 167 deaths as of July 8, 2014 (Chinese Center for Disease Control and Prevention [China CDC], unpub. data). Most cases have occurred in eastern and southern China and have been associated with exposure to poultry at live poultry markets (LPMs). We report a case of human H7N9 virus infection in Jilin Province in northeastern China, an area not contiguous with provinces in which human cases have been previously reported (Figure 1). Furthermore, this case was associated with a small-scale poultry farm, not an LPM.

**Figure 1:**
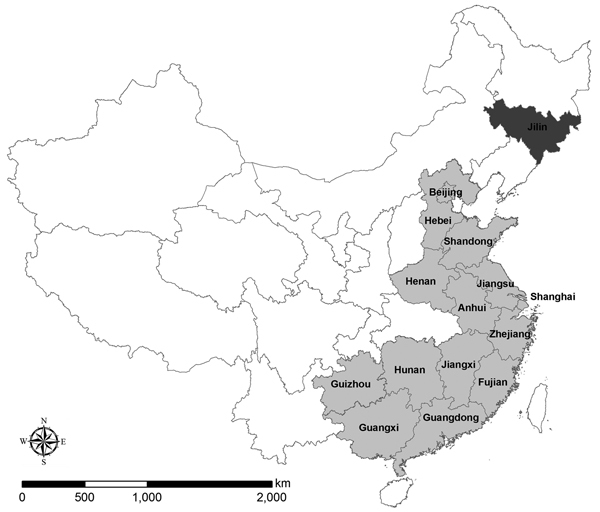
Provinces in China in which human cases of infection with influenza A(H7N9) virus have been confirmed (gray shading). Jilin Province (dark shading), where the case described in this article occurred, shares borders with the Russian Federation and the Democratic People’s Republic of Korea (North Korea).

## The Case

On February 15, 2014, a 50-year-old man, owner of a small farm in Changchun, Jilin Province, experienced an isolated fever (axillary temperature 38.3°C). He had no history of underlying medical conditions. The man sought medical care that day at Jilin University Third Hospital and returned to the hospital on February 16, 17, and 19 with ongoing fever. Radiographic imaging showed evidence of pneumonia; he received intravenous administration of azithromycin and xiyanping, a traditional Chinese medicine. On February 19, the man sought care at Jilin University First Hospital, where throat swab samples were taken. The same day, testing of the samples at the local and provincial Centers for Disease Control and Prevention yielded positive results for influenza A(H7N9) virus by real-time reverse transcription PCR (RT-PCR); China CDC confirmed results the next day. Treatment with oseltamivir (150 mg 2×/d) and methylprednisolone (80 mg 2×/d) was initiated, and oxygen was administered by nasal cannula. The man recovered and was discharged from the hospital on March 7.

The man denied exposure to poultry other than on his farm, including to poultry on other farms or in LPMs. He reported no contact with persons who had similar symptoms before onset of his illness. As part of the case investigation, 68 close contacts of the case-patient were monitored for 7 days; 1 contact had influenza-like illness. Throat swab specimens were collected from this person on days 2 and 3 after symptom onset and tested for influenza A(H7N9) by using real-time RT-PCR; results were negative.

The virus from the case-patient’s specimens was isolated in egg culture and designated A/Jilin/10117/2014 (H7N9) (full sequence available from GISAID, accession no. EPI_ISL_161665). The isolate’s 8 genes were similar to those of the virus A/Anhui/ 02/2013 (H7N9) (GISAID accession no. EPI_ISL_141190); nucleotide/amino acid homology was 99.5%/99.6% for hemagglutinin, 99.2%/98.9% for neuraminidase, 99.3%/99.7% for polybasic 1, 96.4%/98.9% for polybasic 2, 97.9%/99.6% for polymerase acidic, 99.7%/99.6% for nucleoprotein, 97.7%/100.0% for matrix protein, and 99.5%/99.1% for nonstructural protein.

**Figure 2:**
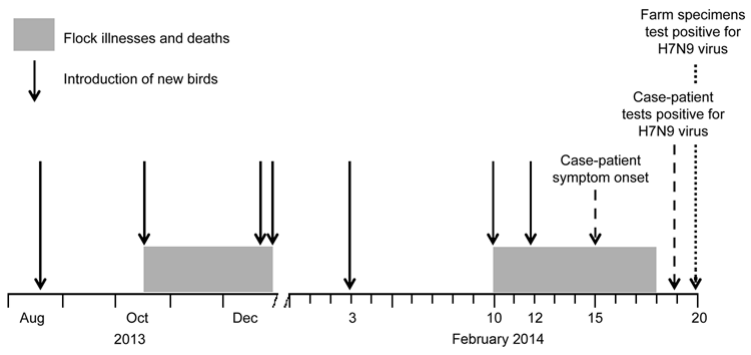
Timeline of introduction of new birds to the farm of the case-patient with influenza A(H7N9) virus infection in Jilin Province, China, 2013–2014. Dates of illnesses and deaths among bird flock on farm, the case-patient’s symptom onset, and confirmed testing results are indicated.

**Figure 3:**
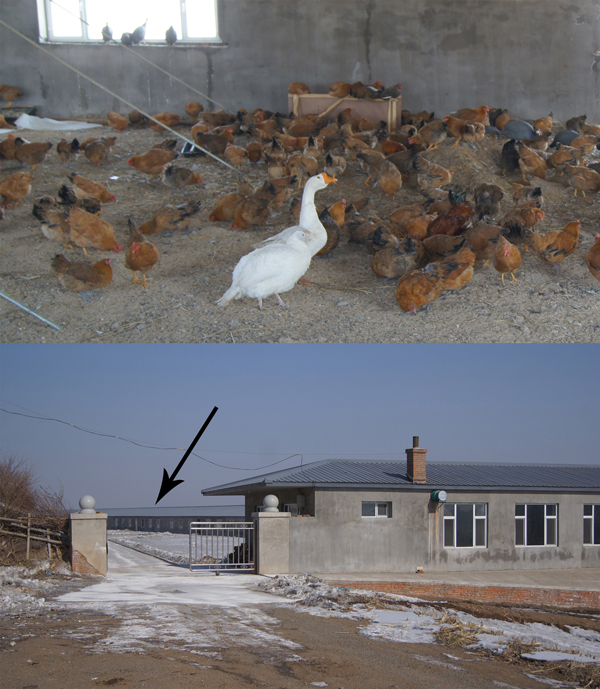
Internal (top) and external views of the warehouse where poultry were housed on the farm of the case-patient who had confirmed influenza A(H7N9) virus infection in February 2014 in Jilin Province, China. Arrow indicates location of the chicken warehouse.

During August 2013–February 2014, the case-patient had introduced 7 groups of birds to his farm, totaling ≈1,100 birds from 8 source farms (Figure 2). The birds were turkeys, guinea fowl (*Numida meleagris*), black-bone silkie chickens (*Gallus gallus domesticus*), local chickens, and a goose. None of the birds received avian influenza vaccines on the case-patient’s farm; previous vaccination histories were unknown. Birds commingled in an egg production warehouse (Figure 3) maintained by the case-patient and by 2 farmers hired in early January 2014. The warehouse was seldom cleaned and never disinfected. The case-patient cared for the birds daily and did not use personal protective equipment.

In October 2013, a group of 280 silkie chickens and guinea fowl was introduced to the warehouse and subsequently exhibited signs of illness; many of these birds died during the next 3 months. A veterinarian reportedly diagnosed air sacculitis and enteritis; testing for influenza A was not performed. By January 2014, all but 4 silkie chickens and 40 guinea fowl had died. More birds were added on February 3, but no further illness was noted until 2 additional flocks, totaling 290 birds, arrived on February 10 and 12. Widespread illness then occurred among the birds, including respiratory and eye secretions, wheezing, and death; >100 birds had died by February 15, when the case-patient’s symptoms began. The Changchun Prefectural Bureau of Agriculture performed necropsies and attributed these deaths to a mixed infection of fowlpox and colibacillosis.

Investigation and sampling at the case-patient’s farm and related suppliers were conducted by local, provincial, and national CDCs. Public health laboratories tested 84 poultry and environmental specimens collected from the farm (Table); 19 samples were positive by real-time RT-PCR for both influenza A(H7N9) and A(H9N2), 1 was positive only for H7N9, and 3 were positive only for H9N2. A total of 374 samples were collected from the 8 source farms, the farms and transport vehicles of the 2 distributors who delivered chickens to the case-patient’s farm, and 56 other local farms. Four specimens from 1 distributor tested positive for influenza A H9 by RT-PCR; neuraminidase subtyping was not performed on these specimens.

**Table:** Specimen collection and results of real-time reverse transcription PCR testing for epidemiologic investigation into source of human influenza A(H7N9) virus infection, Jilin Province, China

Source and specimen type*	Total no. specimens	No. (%) positive results†		
		H7N9 and H9	H7N9 only	H9 only
Case-patient poultry farm				
Poultry feces	25	1 (4)	0	0
Sewage	8	3 (38)	0	0
Environmental swab samples (chicken troughs)	13	7 (54)	0	0
Oropharyngeal samples	20	7 (35)	1 (5)	3 (15)
Cloacal samples	17	0	0	0
Cloacal and oropharyngeal samples	1	1 (100)	0	0
Environmental samples from distributors’ farms and transport vehicles‡	13	0	0	4 (31)
Source farms and other area farms§				
Poultry feces	148	0	0	0
Sewage	32	0	0	0
Environmental swab samples (chicken troughs)	150	0	0	0
Oropharyngeal samples	12	0	0	0
Cloacal samples	19	0	0	0

*One oropharyngeal swab sample from a goose and 1 oropharyngeal swab from a turkey were included in the specimens from case-patient poultry farm; both samples were positive for influenza A(H7N9) and A(H9N2). All other cloacal and/or oropharyngeal swab specimens were from local chickens. †H9-positive samples from the case-patient poultry farm were influenza A(H9N2). H9-positive samples from other sources were not further characterized by subtype. ‡Distributors bought poultry from companies or local farmers (source farms) and then sold the poultry to others, including the case-patient. Poultry was kept at distributors’ farms before resale. Samples were taken from transport vehicles and farms of 2 distributors who transported the chickens to the case-patient’s farm. All positive specimens were from a single distributor: 3 from his transport vehicle and 1 from his farm. §Eight farms that supplied the birds to the case-patient (source farms) and 56 other farms located in the villages of the case-patient and source farms.

## Conclusions

We describe a human case of influenza A(H7N9) virus infection epidemiologically linked to a poultry farm from which samples tested positive for H7N9 and H9N2 viruses. Previous reports of human subtype H7N9 virus infections in mainland China identified a poultry-related exposure for 84% of case-patients, 77% of which were linked to LPMs, but none were linked to farms (China CDC, unpub. data). According to China’s Ministry of Agriculture, almost all positive samples have been from LPMs; H7N9 virus was initially isolated from farms in Guangdong Province in southern China in mid-March 2014 (*2*). Samples collected from a farm in Zhejiang Province were also positive for influenza A(H7N9) virus by real-time RT-PCR, but human cases were not epidemiologically linked to this farm (*3*) .

Unlike avian influenza A(H5N1) virus, which frequently causes severe disease and death in poultry, influenza A(H7N9) virus is a low pathogenicity virus and has not been observed to cause substantial deaths in avian species. Under experimental conditions, birds exhibited no clinical disease after H7N9 virus challenge, although chickens and quail shed high levels of virus (*4*). The poultry on this case-patient’s farm were co-infected with other pathogens, and many died in the week preceding the case-patient’s illness. The introduction of even a low pathogenicity virus into a flock already challenged with other pathogens likely contributed to the rates of illness and deaths.

This case report underscores the importance of highly sensitive assays in detecting H7N9 virus in poultry and their surrounding environments. The virus was detected in a substantial proportion of specimens on the case-patient’s farm (20/84, 24%); in addition, we found that 95% (19/20) of specimens positive for H7N9 virus were also positive for H9N2 virus. The H7N9 virus possesses internal gene cassettes from poultry H9N2 virus, another low pathogenicity virus that circulates widely in poultry in Asia (*3*,*5*,*6*). Co-circulation of H7N9 and H9N2 viruses was reported in LBMs in Hangzhou but not on farms (*3*).

Public health measures taken to contain the outbreak of H7N9 virus infection have thus far focused on LPMs (*7*–*14*), but our findings suggest that small-scale farms may be another source. Inadequate cleaning and disinfecting, a lack of personal protective equipment, and the comingling of bird species found on this farm are common in small-scale poultry farms. These factors enable evolution of novel avian influenza viruses and spread of H7N9 virus. Improved surveillance and biosecurity on farms in China is crucial to containing this outbreak.
